# Editorial: Statistical methods for analyzing multiple environmental quantitative genomic data

**DOI:** 10.3389/fgene.2023.1212804

**Published:** 2023-06-19

**Authors:** Zitong Li, Lucia Gutierrez

**Affiliations:** ^1^ CSIRO Agriculture and Food, Canberra, ACT, Australia; ^2^ Department of Agronomy, University of Wisconsin-Madison, Madison, WI, United States

**Keywords:** genomic selection and prediction, plant and animal breeding, genotype environment interactions, statistical models, dimensional reduction

Phenotypic variation arises from the combined effects of genetic and environmental factors, including interactions between them (Lynch and Walsh, 1998). Studying the relationship between phenotypes, genotypes, and environments using sophisticated statistical models becomes a crucial Research Topic in quantitative genetics (Crossa et al., 2021). Recent advancements in high-throughput genotyping (Hu et al., 2021) and phenotyping (Gill et al., 2022) measurement techniques have enabled the acquisition of large-scale genomic, phenomic, and environmental data by quantitative geneticists. This Research Topic highlights several novel statistical analytical tools that can effectively leverage high-dimensional data to gain a deeper understanding of genotype-environment interactions (GEI) (Elias et al., 2016; van Eeuwijk et al., 2016) and use them to predict phenotype outcomes.

One important research direction in plant and animal breeding is genomic selection or genomic prediction (GP) (Meuwissen et al., 2001), a molecular breeding technique that uses genome-wide datasets to predict the genomic estimated breeding values (GEBV) or genotypic values of individuals for economically important traits. Many quantitative traits, such as yield, have a very complex genetic architecture (Doerge, 2002; Bernardo, 2016). Therefore, incorporating environmental data into the genomic prediction model and properly describing gene-environment interactions (GEI) is crucial to provide promising predictions of individual’s performance. Different strategies have been used to accounting for GEI in GP models and it is still an area of active research (Jarquín et al., 2021). A natural modelling approach includes extending GP mixed models to incorporate a covariance relationship matrix between environments based on phenotypic information (Piepho, 1998; Burgueño et al., 2012; Lado et al., 2016; Malosetti et al., 2016) or environmental covariates (Jarquín et al., 2014). These can be included as either linear or non-linear kernels (Costa-Neto et al., 2020). Models based on observed covariance among environments cannot however predict the performance of individuals in untested environments (Heslot et al., 2014). An alternative to predict the performance of individuals in untested environments is to use environmental covariates in either partial-least squares regressions (Crossa et al., 1999; Monteverde et al., 2019) or for genotype-specific reaction norms in random regression models (Schaeffer, 2004; Buntaran et al., 2021) or P-splines (Bustos-Korts et al., 2021). Due to high availability of environmental covariates and their highly correlated nature, not all environmental covariates are equally informative (Bustos-Korts et al., 2015) and variable selection has been proven useful in improving the performance (Neyhart et al., 2022).

Conventional prediction models, such as reaction norm model (Jarquín et al., 2014) or genomic best linear unbiased prediction model (G-BLUP) for GEI, face particular challenges when used to predict untested lines in new environments. As an improvement, Montesinos-López et al. demonstrated the use of partial least squares (PLS) regression approaches (Boulesteix and Strimmer, 2007) for conducting multiple environmental genomic prediction in 14 real data sets. The PLS method can simultaneously account for G, E and GEI effects for genomic prediction, and the multiple case studies have demonstrated that PLS can provide more accurate prediction compared to conventional GP methods for lines in new environments. Montesinos-López et al. further extended the PLS approach to a multivariate PLS regression that can simultaneously analyse multiple traits and it showed supervisor prediction performance to single trait PLS as well as G-BLUP because the MPLS approach can account for the correlation among traits and can therefore borrow strength from each other during the analysis.

When incorporating more data into a genomic prediction model, the numerical computation for parameter estimation may become infeasible. To overcome this challenge, Manthena et al. evaluated a series of dimensional reduction methods such as random projection, random and deterministic sampling, and shrinkage methods which were applied to reduce the dimension of the SNP data ahead of the multiple environment GP analyses. The study demonstrated that some of these methods were effective not only on reducing the computational cost, but also can maintain and sometimes even improve the predictability. However, the paper also concludes that there is no dimensional reduction approach which can constantly outperform other methods across data sets. Future efforts are needed to develop more robust dimensional reduction methods compile with the genomic prediction. Additionally, dimension reduction can also be conducted guided by linkage disequilibrium (LD) (Slatkin, 2008) or the correlation structure among loci. Jin et al. developed a LD network approach to model the correlation among genome-wide markers and cluster them into LD blocks using an efficient sparse graphical learning approach, and the dimension reduction within each LD block using classical principal component analysis. Interestingly, this approach is initially proposed for studying local adaptation using population genomics (Jones et al., 2012) data, but can also be applicable as a tool for dimensional reduction for GP data.

GP models such as reaction norm model and PLS are able to predict outcomes on the basis of GEI, but they cannot be used to identify genes that are associated with GEI. For the gene discovery purpose, Onogi et al. developed a data driven approach named Environmental Covariate Search Affecting Genetic Correlations (ECGC). The ECGC firstly calculated the genetic covariance between the pairwise environments, and then considered the correlation coefficients as the “trait” in the genome-wide association study to identify significant SNPs associated with the environmental stimuli. The ECGC approach was applied on a large-scale soybean data set, which yielded biological meaningful results.

As a conclusion, this Research Topic collects a series of modern quantitative genomic methods that can effectively analyse large-scale genomic, phenomic and environmental data sets, with the aim to either predict individuals’ outcome of quantitative traits or to identify important genes that are linked to genotype by environment interactions. We are hopeful that these new analytical tools can provide useful additions to the existing quantitative genetic methods for analysing high dimensional biological data sets and can also inspire new research development in this existing research area, especially to meet challenges of big data arising in this post-genomic era.
